# Light‐Responsive Dynamic DNA‐Origami‐Based Plasmonic Assemblies

**DOI:** 10.1002/anie.202014963

**Published:** 2021-02-16

**Authors:** Joonas Ryssy, Ashwin K. Natarajan, Jinhua Wang, Arttu J. Lehtonen, Minh‐Kha Nguyen, Rafal Klajn, Anton Kuzyk

**Affiliations:** ^1^ Department of Neuroscience and Biomedical Engineering School of Science Aalto University 00076 Aalto Finland; ^2^ Department of Organic Chemistry Weizmann Institute of Science Rehovot 76100 Israel; ^3^ Faculty of Chemical Engineering HCMC University of Technology VNU-HCM Ho Chi Minh City 700000 Vietnam

**Keywords:** chiral plasmonics, DNA origami, photoacid, self-assembly, stimuli-responsive materials

## Abstract

DNA nanotechnology offers a versatile toolbox for precise spatial and temporal manipulation of matter on the nanoscale. However, rendering DNA‐based systems responsive to light has remained challenging. Herein, we describe the remote manipulation of native (non‐photoresponsive) chiral plasmonic molecules (CPMs) using light. Our strategy is based on the use of a photoresponsive medium comprising a merocyanine‐based photoacid. Upon exposure to visible light, the medium decreases its pH, inducing the formation of DNA triplex links, leading to a spatial reconfiguration of the CPMs. The process can be reversed simply by turning the light off and it can be repeated for multiple cycles. The degree of the overall chirality change in an ensemble of CPMs depends on the CPM fraction undergoing reconfiguration, which, remarkably, depends on and can be tuned by the intensity of incident light. Such a dynamic, remotely controlled system could aid in further advancing DNA‐based devices and nanomaterials.

## Graphical abstract

The spatial configuration and optical properties of non‐photoresponsive DNA‐origami‐based plasmonic assemblies can be controlled with light using a photoresponsive medium. Upon exposure to visible light, the medium's pH decreases, inducing the formation of DNA triplex links in the plasmonic assemblies, leading to their spatial reconfiguration, which can be reversed by turning the light off.

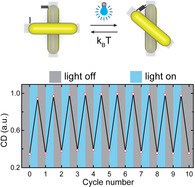

DNA nanotechnology utilizes the specificity and programmability of Watson–Crick base pairing for assembling DNA molecules into complex structures.^[1]^ In particular, the DNA origami technique provides a versatile approach for fabricating almost arbitrarily shaped three‐dimensional nanoarchitectures with high precision and yields.[^2^, ^3^] Recently, DNA‐origami‐based fabrication has evolved toward realizing devices that can undergo a controlled structural reconfiguration.^[4]^ Such dynamic devices hold great promise for use in drug delivery,^[5]^ sensing,[^6^, ^7^, ^8^] nanophotonics,[^9^, ^10^, ^11^] nanorobotics,[^12^, ^13^] and more. Controlled actuation of DNA‐origami‐based structures has been achieved using toehold‐mediated strand displacement reactions,^[14]^ light,[^15^, ^16^, ^17^] temperature,[^18^, ^19^, ^20^] electric[^12^, ^21^] and magnetic^[22]^ fields, ion concentration,[^20^, ^23^] and pH.[^24^, ^25^, ^26^] Light is a particularly interesting external stimulus for inducing dynamic responses within DNA‐based self‐assembled molecular systems.^[27]^ It is a ubiquitous and clean energy source that can be controlled remotely with high spatial and temporal resolution and its characteristics (e.g., wavelength, intensity, and polarization) can be optimized for a specific purpose. To date, the light‐responsive reversible spatial reconfiguration of DNA‐origami‐based assemblies has relied on the covalent incorporation of azobenzene moieties into DNA strands.[^15^, ^16^, ^17^] Although azobenzene photoisomerization has been widely used in the context of stimuli‐responsive materials,[^28^, ^29^, ^30^] the functionalization of DNA strands with azobenzene remains time‐consuming and expensive.^[31]^ Furthermore, light‐based actuation of DNA‐azobenzene structures is relatively slow (the typical time constants are in the tens of minutes range) and usually it requires operating at an elevated temperature (≈40 °C).[^15^, ^32^]

Recently, spiropyran (SP) and its derivatives have emerged as attractive building blocks for constructing artificial dynamic self‐assembled structures and materials.[^33^, ^34^, ^35^, ^36^] In particular, merocyanine‐based photoacids have been used as light‐responsive regulators for generating dynamic assemblies from intrinsically non‐photoresponsive components.[^37^, ^38^, ^39^, ^40^, ^41^] Despite the numerous advantages of spiropyran and its derivatives as light‐responsive molecular switches, their use in the context of DNA nanotechnology has thus far not been demonstrated. Here, we combine the unique properties of a merocyanine‐based photoacid[^42^, ^43^] (MCH^+^) with the pH‐dependent formation of DNA triplexes^[44]^ and the strong chiroptical responses of DNA‐based chiral plasmonic assemblies.^[45]^ Through this combination we can realize a dynamic plasmonic system with remote, light‐controlled modulation of chirality. Importantly, our strategy does not require that the plasmonic assemblies are covalently modified with light‐responsive units; instead, the response to light is “encoded” with the photoresponsive medium.

The design of our system is illustrated in Figure 1. Chiral plasmonic molecules (CPMs) were dispersed in an aqueous solution of MCH^+^. Blue light illumination promotes the ring‐closing reaction of MCH^+^ accompanied by the release of H^+^ (Figure 1 A).^[42]^ The reaction is reversible, i.e., when light is turned off, the closed‐ring SP isomer recaptures H^+^ as it undergoes ring‐opening to regenerate MCH^+^. The release and recapture of H^+^ leads to a decrease and increase of solution pH, respectively. We used this pH modulation to control the state of pH‐sensitive DNA triplex‐based “locks” composed of a 20‐base‐pair duplex and a 20‐base single‐stranded DNA (ssDNA) (Figure 1 B). Protons dissociated from MCH^+^ can protonate the cytosine bases in the ssDNA part of the lock, which leads to triplex formation and closure of the lock through sequence‐specific parallel Hoogsteen interactions.[^44^, ^46^] The pH‐responsive triplex locks were incorporated into CPMs consisting of two gold nanorods (AuNRs) assembled on reconfigurable DNA origami templates (Figure 1 C). The CPMs were designed to adopt the right‐handed spatial configuration (with a ≈50° fixed angle between the rods) when the triplex locks are closed and the relaxed configuration (without fixed angle between the rods) when the locks are open. In other words, the closed state of the triplex locks and thus the right‐handed configuration of CPMs correspond to the metastable state, with the lock spontaneously opening and the CPMs adopting the relaxed configuration upon ceasing light irradiation.

**Figure 1:**
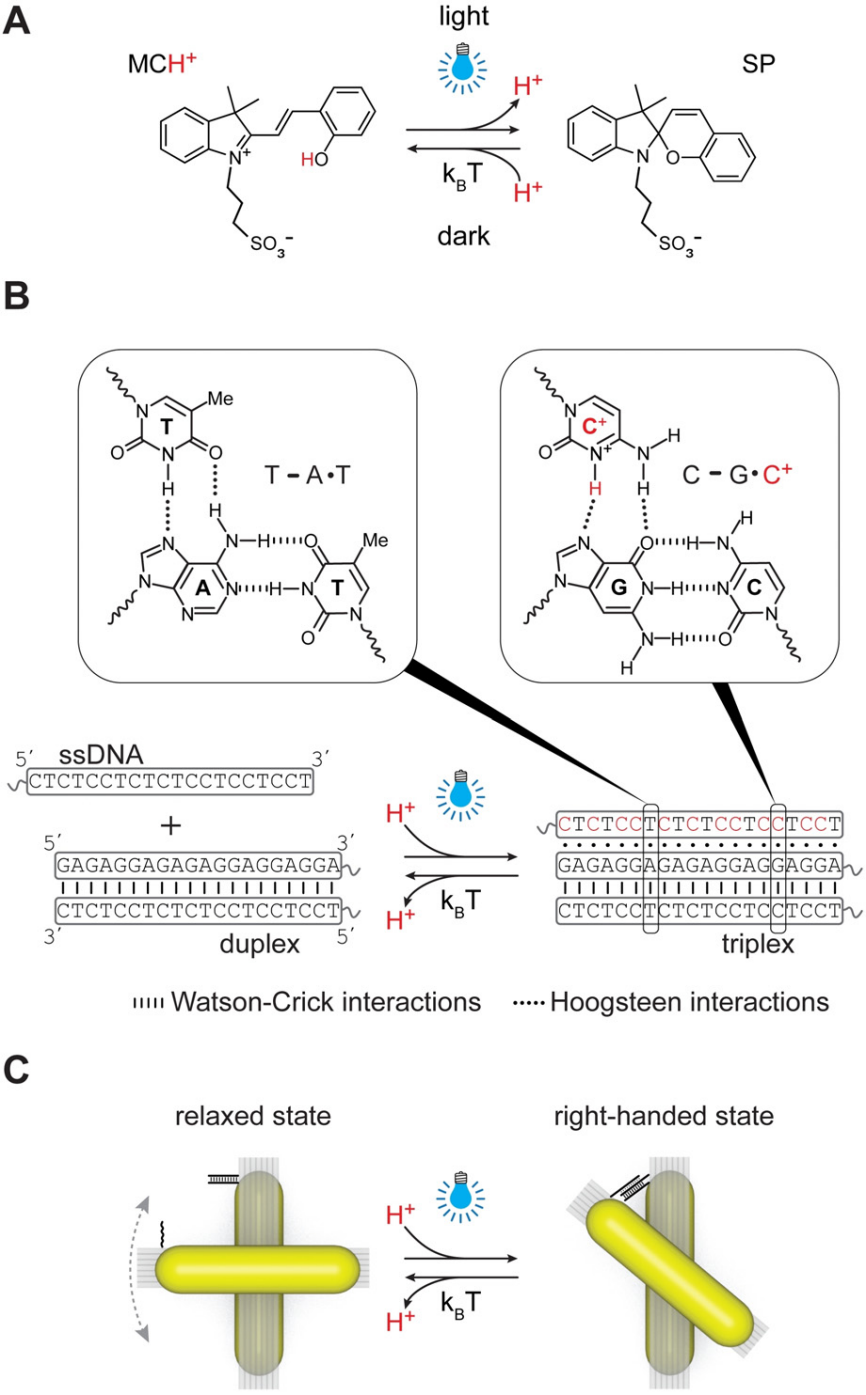
Schematic illustration of the light‐controlled modulation of plasmonic chirality. A) Under blue‐light illumination, MCH^+^ undergoes a photochromic reaction accompanied by the release of H^+^. The reaction is reversible and H^+^ is recaptured when the light is turned off. B) Release of H^+^ leads to decreased pH, which, in turn, affects the state of the pH‐responsive triplex DNA locks. C) The DNA locks are incorporated into CPMs, which can be switched between the relaxed and the right‐handed states by opening and closing of the locks.

Figure 2 shows a representative transmission electron microscopy (TEM) image of CPMs (for additional images, see Figure S3) and their pH‐dependent CD responses. The design and fabrication of CPMs were adapted from previous studies.[^15^, ^24^, ^45^] In short, reconfigurable DNA origami structures consisting of two 14‐helix bundles (80 nm × 16 nm × 8 nm) linked in the middle by two ssDNA crossovers (Figures S1 and S2) were used as templates for the assembly of two AuNRs (25 nm × 62 nm). Pairs of AuNRs constitute CPMs with a circular dichroism (CD) response that is strongly correlated with the spatial configuration.^[47]^ Owing to the high affinity of AuNRs to the TEM grid, the CPMs appear in TEM images as pairs of AuNRs lying side by side^[45]^ (Figure 2 A and Figure S3). The relative content of thymine‐adenine‐thymine (TAT) triplets within the pH‐responsive locks of CPMs can be used to tune the switching of CPMs over a specific pH window.[^24^, ^46^] Previously demonstrated pH‐responsive DNA‐origami‐based CPMs utilized DNA triplexes with TAT content varying between 50 % and 80 %, which could be operated at pH values between 6 and 9.[^24^, ^48^] On the other hand, MCH^+^ has been typically used to modulate the pH of aqueous solutions at pH values below 6.[^38^, ^42^, ^49^] To achieve compatibility between the light‐regulated pH modulation and the switching of pH‐responsive locks, we utilized DNA triplexes with a TAT content of 40 % (Figure 1 B and Table S4). CPMs with 40 % TAT locks exhibited a strong dependence of chiroptical responses on pH in a range between 5.5 and 7 (Figure 2 B). Next, we carefully screened solution compositions to ensure the stability of DNA‐origami‐based CPMs as well as strong pH modulation. Buffer‐free aqueous solution consisting of MCH^+^ (1 mm), 0.8 % DMSO, 500 mm sodium chloride (NaCl), 1 mm sodium bicarbonate (NaHCO_3_), and 0.02 % sodium dodecyl sulfate (SDS) was identified as the ideal medium for the light‐controlled MCH^+^‐mediated actuation of CPMs. Divalent Mg^2+^ ions typically used in DNA origami buffers were avoided because they pose a hindrance to the MCH^+^ photochromic reaction dynamics.^[50]^ DMSO was used to increase MCH^+^ solubility from the water solubility limit^[51]^ of ≈0.2 mm. A relatively high NaCl concentration was required for the stable formation of the triplex DNA locks. SDS surfactant was added to stabilize the CPM structures. NaHCO_3_ was used to adjust the solution pH to ≈6.7.

**Figure 2:**
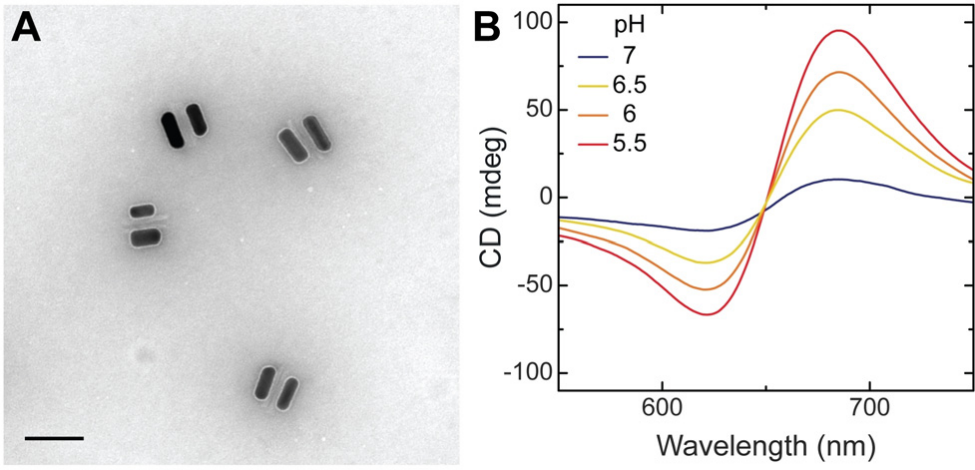
Microscopic and spectroscopic characterization of DNA‐origami‐based CPMs. A) TEM image of CPMs. Note that the observed structures are determined by the nanorods’ tendency to maximize interactions with the underlying TEM substrate and are not representative of the solution structures (Figure 1 C and Figure S2). Scale bar, 100 nm. B) CD spectra of CPMs in solutions of different pH values (adjusted manually).

First, we explored pH modulation of the photoresponsive MCH^+^‐containing solution upon exposure to blue light. An LED with an emission wavelength of 415 nm and a tunable power was used as a light source. When the light was turned on (intensity=1.7 mW cm^−2^), the photoisomerization of MCH^+^, accompanied by H^+^ release, led to a pH drop of ≈1.2 units within 5 minutes (Figure 3 A). This process was reversible, i.e., after 5 minutes in the dark, the released protons were recaptured, and the pH returned to ≈6.7. The pH switching could be repeated for at least ten cycles without any apparent signs of fatigue (Figure 3 B). Next, we dispersed CPMs modified with pH‐responsive DNA triplex locks (40 % TAT content) in the photoresponsive solution (Figure S4) and investigated the light‐induced modulation of CPM chirality. For the optical characterization of CPMs, we utilized cuvettes with three windows, which allowed for simultaneous illumination of the solution and measurement of time‐dependent CD responses at 680 nm (Figure S5). We used a longpass filter with a 455 nm cut‐on wavelength before the CD detector to eliminate the possible interference of the blue light stimulus with the CD measurements. At the initial pH of the solution of ≈6.7, the CPMs were predominantly in the relaxed configuration (Figure S6) and the sample exhibited a low level of CD response (Figure 3 C). However, when the blue light was turned on (intensity=1.7 mW cm^−2^), CPMs started to adopt a closed, right‐handed configuration (Figure S6) with a much stronger CD response. In the dark, the structures returned to the original state with a predominantly relaxed configuration. The typical time constants for the opening/closing kinetics of the pH‐dependent triplexes and the DNA‐ origami‐based CPMs are in the milliseconds^[46]^ and seconds^[24]^ range, respectively. Hence, the light‐induced CPM reconfiguration followed similar kinetics as pH modulation with characteristic switching times of several minutes (Figures 3 A and C). As with the pH modulation, the switching of CD responses could be repeated for at least ten cycles (Figure 3 D). CPMs modified with pH‐insensitive locks exhibited same levels of CD signals under light illumination and in the dark (Figure S9). The switching of the CPMs’ optical response, depicted in Figure 3 D, resembles the typical behavior of switchable molecules actuated between two equilibrium states by modulating the environmental conditions, such as pH, temperature, and ion concentration.^[4]^ An important distinctive feature of our approach is that under continuous light illumination, the system adopts a steady out‐of‐equilibrium state; the CPMs quickly return to the equilibrium (relaxed) configuration upon turning the light off. Thus, the advantage of our system is that it can be reversibly operated using only one type of stimulus (visible light).

**Figure 3:**
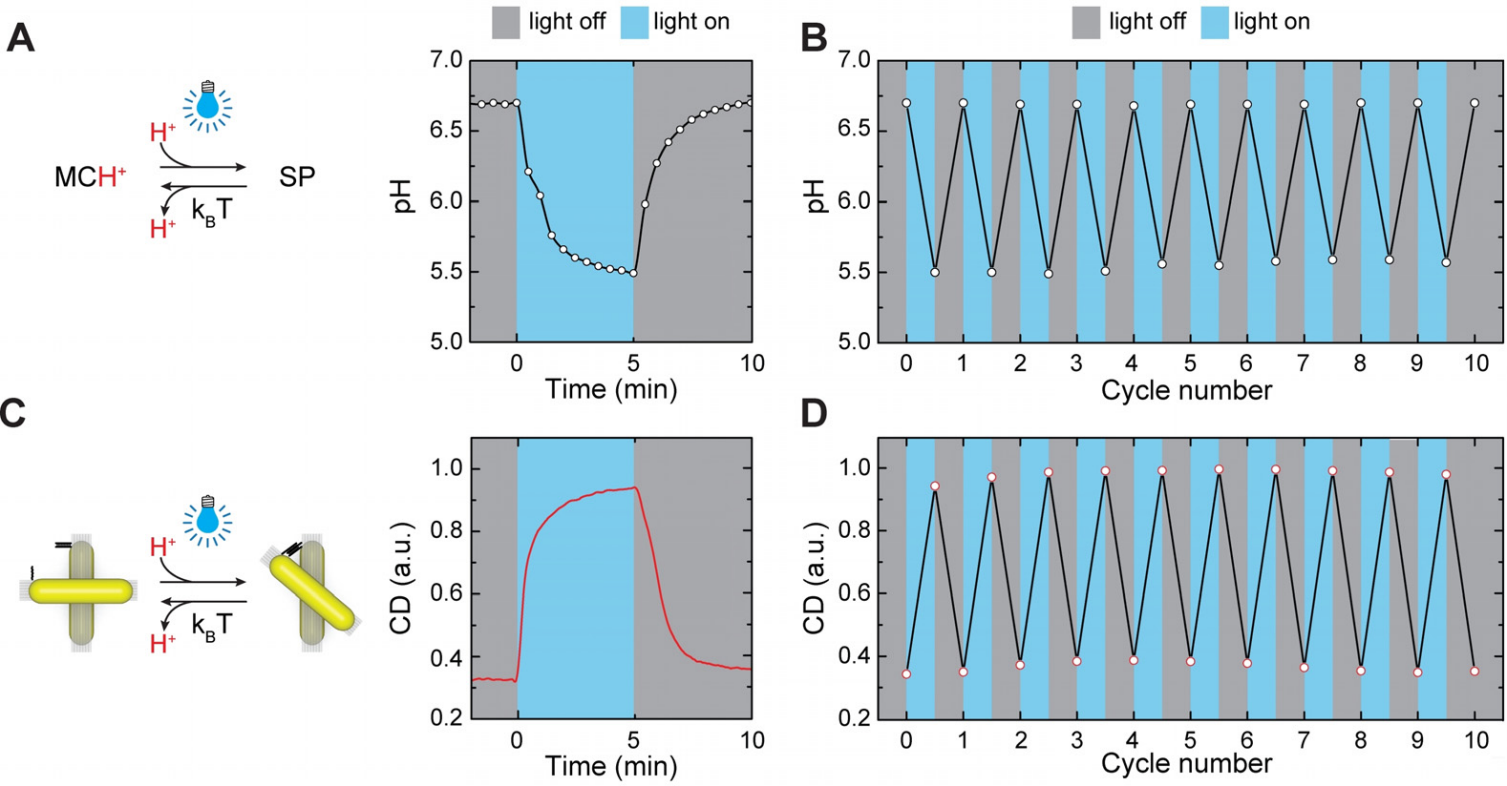
Reversible light‐controlled switching of photoresponsive medium and CPMs. A) Characterization of pH modulation under light illumination (intensity 1.7 mW cm^−2^) and in the dark. The pH reaches a steady state within ≈5 min after the light is turned on or off. B) Reversible switching of pH over ten cycles. C) Characterization of CD modulation under light illumination (intensity 1.7 mW cm^−2^) and in the dark. D) Reversible switching of CD over ten cycles.

Encouraged by the excellent reversibility of the light‐induced switching of the optical response of the CPMs, we investigated whether the amplitude of the CD response could be controlled by tuning the intensity of the incident light. Figure 4 shows the reversible switching of CPMs’ optical response between four different values using four different light intensities (*I*_0_=0 mW cm^−2^, *I*_1_=0.21 mW cm^−2^, *I*_2_=1.3 mW cm^−2^, and *I*_3_=1.7 mW cm^−2^). Under each light intensity, the CD response reached a distinct steady state within ≈5 min (for pH switching under illumination with different intensity, see Figure S10). Furthermore, the switching was completely reversible. These results highlight the excellent dynamic control over the structural and optical properties of DNA‐origami‐based plasmonic assemblies.

**Figure 4:**
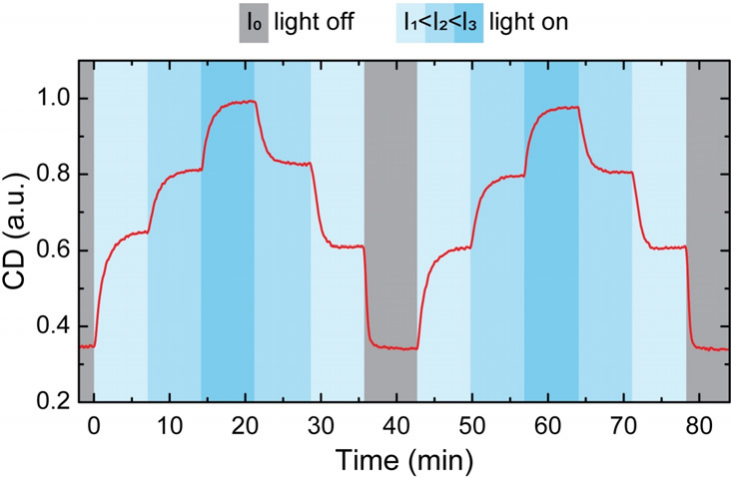
Intensity‐dependent modulation of chirality. Here, the ensemble of CPMs adopted four different steady states with distinct CD levels, depending on the intensity of the incident light. The switching between states took ≈5 min.

In conclusion, we developed a novel approach for the light‐controlled manipulation of DNA‐origami‐based plasmonic assemblies. Our approach combines several unique features. The plasmonic assemblies themselves are not sensitive to light; the photoresponse is implemented through coupling to a photoswitchable environment. The light‐induced switching of plasmonic optical responses is reversible and can be performed numerous times without any apparent signs of fatigue. The degree of chirality in the ensemble of CPMs and the amplitude of CD responses can be controlled by the incident light intensity. Our system represents a unique example of light‐controlled self‐assembly with easily regulated steady out‐of‐equilibrium states. Importantly, our approach is not limited to plasmonic assemblies and could readily be adopted to a wide range of DNA‐based devices and circuitry. We anticipate that our results will further advance the development of stimuli‐responsive molecular and colloidal assemblies with functionalities tailored to individual and specific needs.

## Conflict of interest

The authors declare no conflict of interest.

## Supporting information

As a service to our authors and readers, this journal provides supporting information supplied by the authors. Such materials are peer reviewed and may be re‐organized for online delivery, but are not copy‐edited or typeset. Technical support issues arising from supporting information (other than missing files) should be addressed to the authors.

Supplementary
