# Supplementary material for: Light‐Responsive Dynamic DNA‐Origami‐Based Plasmonic Assemblies
Source: Angew Chem Int Ed Engl. 2021 Feb 16;60(11):5859–63. doi: 10.1002/anie.202014963 (PMC7986157; doi:10.1002/anie.202014963)
Supplement: Supplementary file 1 — Supplementary [file ANIE-60-5859-s001.pdf]

Angewandte Chemie
Eine Zeitschrift der Gesellschaft Deutscher Chemiker

Supporting Information

Light-Responsive Dynamic DNA-Origami-Based Plasmonic Assemblies

*Joonas Ryssy, Ashwin K. Natarajan, Jinhua Wang, Arttu J. Lehtonen, Minh-Kha Nguyen, Rafal Klajn, and Anton Kuzyk**

anie_202014963_sm_miscellaneous_information.pdf

This PDF file includes:

Experimental section with details of design, fabrication and, characterization of DNA-origami-based chiral plasmonic metamolecules (CPMs)

Figure S1. Scaffold/staple layout of the DNA origami structures

Figure S2. TEM micrographs of the DNA origami structures

Figure S3. TEM micrographs of the CPMs

Figure S4. TEM micrographs of the CPMs in photoresponsive medium before and after illumination

Figure S5. Schematic representation of the experimental measurement setup

Figure S6. Histograms of the acute angle between two bundles of DNA origami templates

Figure S7. Examples of the acute angle characterization in buffers with pH 6.7 and 5.5

Figure S8. Examples of the acute angle characterization in photoresponsive medium with and without illumination

Figure S9. CD responses of CPMs with pH-insensitive locks

Figure S10. Intensity-dependent modulation of the photoresponsive environment pH

Table S1. Sequences of staples for assembly of DNA origami structures

Table S2. Sequences of the pH-sensitive DNA locks

Table S3. Thermal annealing temperatures and times for DNA origami assembly

Author contributions

Experimental Procedures

Fabrication of DNA origami structures

The design of the DNA origami structures was adopted from previous studies.[1–3] Reconfigurable DNA origami structures consisting of two 14-helix bundles (80 nm × 16 nm × 8 nm) linked in the middle by two ssDNA crossovers were used as templates for the assembly of two gold nanorods (AuNRs) with an average size of 25 nm × 62 nm. For the AuNRs assembly, 36 staples (18 on each bundle) were modified with additional A_{10} extensions at the 3' end. Scaffold/staple routing of the DNA origami template is shown in Figure S1. The sequences of all staple strands are provided in Table S1. Sequences of the pH-sensitive DNA locks are provided in Table S2. The origami structures were prepared by thermal annealing (Table S3). The reaction mixture contained 10 nM p7560 scaffold (tilibit nanosystems GmbH), 100 nM of each staple (Fisher Scientific), 20 mM of $MgCl_2$, and 1× Tris EDTA (TE) buffer. After assembly, the excess staples were removed using spin-filtering method[4] and Amicon Ultra-0.5 centrifugal filter units (cutoff: 100 kDa). Representative TEM images of DNA origami structures after fabrication and purification are shown in Figure S2.

Gold Nanorod Synthesis

Gold nanorods (AuNRs) were fabricated using the previously established protocol[2,3] with minor modifications. First, ~2 nm CTAB-capped gold seeds were prepared. 0.5 mL of $HAuCl_4$ (0.5 mM) was mixed with 0.5 mL of CTAB (0.2 M) at room temperature. 100 µL of cold $NaBH_4$ (6 mM, 4 ºC) was added quickly to the solution while it was stirring at 1200 rpm. The solution was mixed for 2 min and incubated at 30 ºC for 30 min before use. Next, AuNRs were synthesized from the seeds as follows. The gold growth solution was prepared by gently mixing 15 mL of CTAB (0.1 M) and 2,6-dihydroxybenzoic acid (15.6 mM), 0.54 mL of $AgNO_3$ (4 mM), and 15 mL of $HAuCl_4$ (1 mM) in a 250 mL round bottom flask at 30 ºC. After which, 96 µL of L-ascorbic acid (64 mM) was injected and stirred at 1200 rpm for 30 s. 6 µL of the prepared CTAB-capped gold seed solution was added into the mixture, which was then stirred for 30 s and kept undisturbed at 30 ºC for 18 h. The synthesized gold nanorods were washed three times with CTAB (0.1 mM) by centrifugation at 9500 rcf for 10 min. The rods were dispersed in 0.1 mM CTAB and stored at 4 ºC.

Functionalization of Gold Nanorods with DNA

The procedure for preparation of DNA-functionalized AuNRs (AuNRs-DNA) was adapted from previously established protocol[3] with minor modifications. Thiol-modified DNA strands (SH-5' T_{16}, 1 mM) were pre-incubated with tris(carboxyethyl) phosphine hydrochloride (TCEP, 14 mM) for 1 h before mixing with 10 nM AuNRs dispersed in water with 0.05% SDS. The AuNR:DNA ratio was 1:14000. pH of the solution was adjusted to 2.5-3.0 with 1 M hydrochloric acid. The mixture was incubated for 1 hour at room temperature with shaking at 400 rpm. 1 M solution of sodium chloride (NaCl) was added to the AuNRs-DNA sample until the concentration of 0.5 M was reached, followed by additional shaking of the solution for 4 hours. The pH of the solution was readjusted to 7.5-8.0 with a 10× TBE buffer. After overnight incubation, the AuNRs-DNA sample was washed in a 0.5× TBE buffer containing SDS (0.1%) for four times by centrifugation at 7000 rcf for 30 min. The concentration of AuNRs-DNA was estimated using the absorbance value at the longitudinal plasmonic mode resonance (650 nm) and extinction coefficient of $3.8 \cdot 10^9$ M^{-1} cm^{-1}. AuNRs-DNA were stored at 4 ºC until use.

Assembly of DNA-origami-based chiral plasmonic metamolecules (CPMs)

The assembly procedure was adopted from previously established protocol.[3] $MgCl_2$ was added to AuNR-DNA solution (final $MgCl_2$ concentration 10 mM). AuNRs-DNA were mixed with DNA origami templates at 15:1 ratio and annealed overnight in a thermoshaker (Eppendorf, ThermoMixer) from 40 ºC to 20 ºC. A 0.75% agarose gel with 12 mM $MgCl_2$ was used to purify the assembled CPMs (3h, 80V, ice bath). The CPMs band was cut and extracted using a gel extraction filter (Millipore, 42600). TEM images of CPMs after fabrication and purification are presented in Figures S3. Prior to dispersion in photoresponsive medium, CPMs were washed three times by centrifugation (5 min, 3k rcf) in buffer-free aqueous solution containing 500 mM NaCl and 0.02% SDS.

Photoresponsive environment.

Photoacid MCH^+ was prepared following the literature procedure.[5] For the preparation of the photoresponsive medium, 1.28 mg photoacid was diluted in 830 µl of 1% DMSO aqueous solution. Buffer-free aqueous solution consisting of MCH^+ (1 mM), 0.8% DMSO, 500 mM NaCl, 1 mM sodium bicarbonate ($NaHCO_3$), and 0.02% sodium dodecyl sulfate (SDS) was identified as the ideal medium for the light-controlled MCH^+-mediated actuation of CPMs. The pH of the solution in the dark was ~6.7. The light-induced pH modulation of the photoresponsive environment (Figures 3A, B in the main text) was measured using Mettler Toledo SevenCompact S220 pH meter equipped with InLab Ultra-Micro electrode.

CD characterization of CPMs

Chiroptical properties of CPMs were characterized using Jasco J-1500 Circular Dichroism spectrometer equipped with Jasco EXPM-531 detector. The time-depended CD responses were measured at 680 nm. All the CD measurements were performed using QS High Precision Cell cuvette with three windows, light path of 3 mm, and a center height of 8.5 mm (Hellma, 105-251-85-40). For the light-induced reconfiguration of CPMs, an LED with an emission wavelength of 415 nm was used as a light source (Thorlabs M415L4). The LED emission was collimated using an adjustable adaptor (Thorlabs SM2F32-A). A longpass filter with cut-on wavelength of 455 nm (Thorlabs FGL455) was placed before the CD detector to eliminate possible interference of the LED emission with the CD characterization (Figure S6).

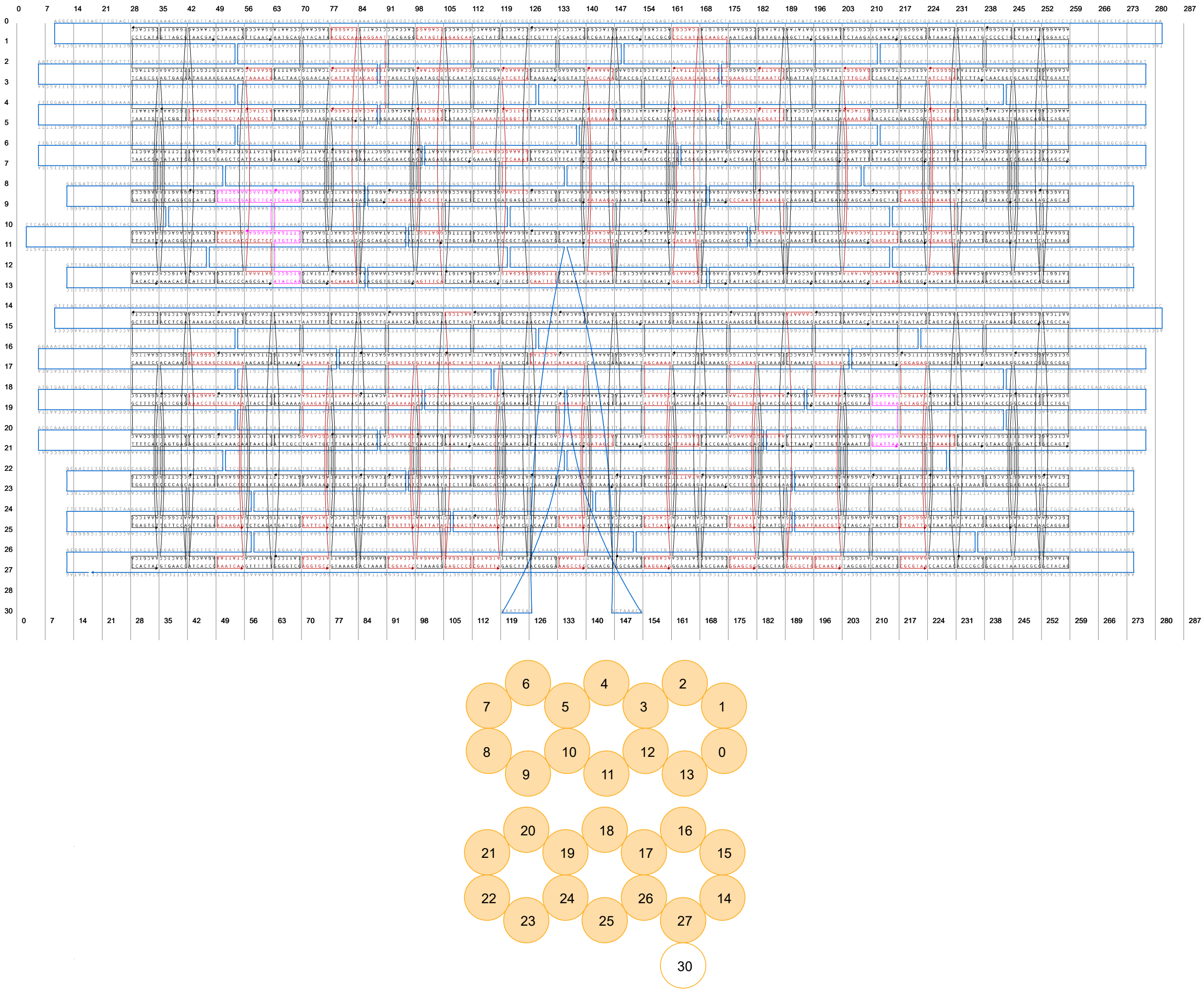

Figure S1: Scaffold/staples layout of the DNA origami structures.

SUPPORTING INFORMATION

Table S1: Staple sequences of the DNA origami template. The colors of the sequence in the table correspond to the staple colors in Figure S1.

Staples	Length (nt)	Start	End
AATAATTTCAGCGGCTACGAATACACTA	28	4[34]	13[34]
AGGTCCGGATATTCTGACGAGGATGGTT	28	8[90]	6[77]
AGTAATCTTTTAGTCTAGAAAAAGCCTA	28	24[146]	18[133]
GAAATACCATTGCATTAAGCAGCCTTTA	28	25[161]	16[161]
TATTAATCAAGGCATAAAAATTTTTAGA	28	26[146]	16[133]
TCATAGGTCTGAGAAAAACATCACGAAT	28	16[104]	14[91]
TCTTTAATGCGCAGTTAGAGCCGTAAAA	28	22[146]	23[146]
GTAGCAAATCGGCCCCATAAATTAATGC	28	25[203]	17[216]
GGGGTGCCAATTCCAACGTCACCACTAC	28	18[34]	27[34]
AAACACTTGAGTTTGTTAGCGTAACGAT	28	13[35]	1[48]
AGCCTAACAATTTTTTCAACCAGGTGGC	28	4[223]	13[223]
GAGCTAAGCTTTCCGCGATCGGGCGATT	28	25[245]	16[245]
GCGAGAGCCAGACGCACCCTCAGAACCG	28	2[139]	0[126]
GATTACAAACAGTACTTCTGTAAATCCC	28	26[62]	16[49]
CGTTGGGGGAACAATTGTATCGCGCGAA	28	4[76]	13[76]
TGAATCCCTGCGGATAGCTCATAACAGT	28	4[118]	13[118]
CTGAGTAGGTAGCTCAGGGTTTTTTCAA	28	26[230]	16[217]
GGCCTTCAAGAGTCACGGTAACTGGAGC	28	23[203]	18[203]
CGAACGTATGATACATGCAATGCCTGAG	28	27[140]	15[153]
ATCGATATAGAGCCGAGGCAGGAATGGAAACTAAC	35	9[245]	4[238]
TTTAGCGCCGGTATGAGGGTTGATATAATTAGCAA	35	2[202]	13[195]
TAAGATCAAGCCAACGCTCATAGCCGAAAGAACTG	35	12[174]	12[182]
CTAAAGGCGGTGGGAGCGATAGCTTAGAATCAAAA	35	27[98]	16[105]
ATCCTTTGCCCGACCACGACCGGGACATCTATTAG	35	25[140]	22[147]
CTAAACAATGTACCGTAACACCATCTTTAATACGT	35	1[49]	12[42]
CCTTCTGACCTGAAAAATGGAAATACCGACCGTAA	35	23[175]	19[195]
CTAATAGCTTTTTCAGTGCCATATCTGGTCGAACT	35	18[132]	21[139]
TAAATTGGGCTTGAAAACACCAGAACGACTCCAAC	35	6[97]	8[91]
GTAAAGCCCTTGAACCTTAGAATCCTTGGACTACC	35	27[77]	16[84]
CATTAAGAAAACGATTAATCATAAGGGAACAGGAT	35	5[84]	9[90]
AATAAACTTGCTATACGGAATACGTAGAAAATACA	35	4[202]	13[209]
AATATACTTTGAATACCAACACCTTGCTTTTAACG	35	22[83]	22[91]
TCGATGATGTCCATCACGCTGAGTAATTCGCGTCT	35	19[196]	23[202]
CGGGAGGAATCAGACCGGAATAGGTGTATTCTCCT	35	2[181]	13[174]
AATAGATATGCAGAACGCGCCTCTGTCCAAGTACC	35	6[153]	9[160]
CAATATACGGAATTTGAACCTCCGGCTTACATTTA	35	25[77]	18[84]
TTTTGTTCGCCATCAAAAACGTAAGAATCCTAAAC	35	21[196]	21[188]
TACCGCGACTCAGGAGGTTTATTAGTTTGACCATT	35	1[154]	13[160]
ATAGGAAAAAAATTCGATTGTATAAGCAAATATTTAGTTAATA	42	22[209]	21[195]
ACTGAACACAAGAATTGAGTAAAGTAATTGACGGGAGAATTA	42	7[175]	7[174]
ATTTAATTAAAACATACCGAACGAACCAACGTGGCACAGACA	42	19[168]	22[168]
GTATTTCATCTTTAGGAATTGAGGAAGGGAACCTCGAAAAAT	42	24[111]	20[98]
GTTTTTTTCACATACGTCGCTATTAATTAACCTTGCATAAAT	42	27[56]	17[69]
CTTATCATTCCAAATTACCCTGACTAACCCAACATGCATCAT	42	4[139]	11[139]

GGATAGGTGCATCTCCAGCTTTCGGTTGATAATCAGCGCATC	42	22[251]	21[237]
TCAGCTAAAGTCCTGATTATAGTCAGAAATCGCGTTTCATGT	42	7[140]	7[139]
TTGCGTACCTACCAATGAAACAAACATCATGTTACAAAATCG	42	23[77]	20[77]
TTACATCGGGAGAGAATCCTGTTTAAAACAGAAATGTACCTT	42	22[62]	22[63]
ACCCAAACAAAGTTTAAGAAACAAGAAACAGAGAGATAACCC	42	12[195]	8[182]
CAACGGCGCCAAAGACGCAAAGACACCATTAGGATCCTATTT	42	3[238]	1[251]
ATCAATAACTCATCGTCAATAACCTGTTATACAAATTCTTAC	42	4[160]	11[160]
TTTCAACTAATGTGCCTGGAGTGACTCTGGCGAGAAAAACGT	42	16[160]	26[147]
TGCATTAACGGGCAACAAACAATAACGGATTCATTTCCCAGC	42	20[48]	20[49]
AAACGGGTAAAAATCGGTGTACAGACGGATCGGTTTGCGAAT	42	11[35]	4[35]
AGAGAGGTCGTTAGGCGCTTAATGCGCCGCGCACGACGGCCA	42	17[238]	15[251]
GGTGAATGGTCGCTGAGCTCATTCAGTGATCATTGTGTTCGA	42	6[48]	6[49]
TAACCGAAAGGCCGGACAGCATCCAGGCGCATAGGCTTGCAG	42	7[28]	8[42]
GCAAGGAAAGAATTATAATTATAATTTCAGTATTACTAAAAC	42	16[153]	21[153]
CATATTTGAATATAAGACGACGACAATAAACAATAAGCCAGT	42	10[153]	9[139]
TGATTCCCCACCCTATAACCCTCGTTTAGCTTTTGTAAGAAC	42	13[119]	3[132]
AAGTTGGTAAAACGACTTAAGTGTCCTTACCCGCCAACTTGC	42	16[244]	26[231]
GAAAGACGCGAACCCCTTTTGATGAGGCATTTTCGATTCGAG	42	7[112]	8[126]
GAGCTTGTGCCATCGCTGAGAAGCATATATTTTAACGACAGT	42	27[119]	14[133]
GTAATAACATCATCTTTTATAATTAAATGGTGTAGATTCAGC	42	25[224]	22[224]
GCATGATTATTACGCAGTATGGTATAGCTATAGAAGGCTTAT	42	12[181]	1[195]
TCAGTTGATACATATAGCAAGCCCAATAGGAACCCTTCAACT	42	2[76]	1[62]
GTGAACCACTCTGAACCTCGATAAAGACGGAGGATAATCATT	42	27[35]	14[49]
ATCAACACAATCAACGCTGAGAGCCAGCAGAACGCTTTAGAA	42	22[125]	24[112]
TAATTTCTTTAAGATGACCAATTAGCCGGAACGAGCGGAGAT	42	6[76]	12[77]
CTTGCCCATTACCCTAATCTTGACAAGAACCGAACACTGGCT	42	7[70]	5[83]
AATCTACAAAGGCTCATATGTACCCCCGGAAGTGTAGAGCGG	42	18[244]	25[244]
CCACCACAGTGCTGCAGTCACGACGTTGGTAACGCATTTTTG	42	27[224]	17[237]
AGTAAAATACGAGGCACCACCCTCATTTACACGGTGTCTGGA	42	2[97]	13[97]
AATGCCAAGTGAGATTCCAGACGTTAGTCCTCATACGTCACC	42	12[41]	0[28]
AACATATTCAGTACATAAACAGTTAATGGTTTTAAATCTTAC	42	13[224]	3[237]
CTATTTAAGCGAAACGCCAGCCTACATTCATTGGCATAGAAC	42	14[174]	23[174]
AGGCAAAGAGGTCAATATAATGCGCTGAAAAGGTGGTAATTT	42	10[125]	10[126]
AGTTTTGCACTATCCAGAACCGCCACCCTTCCATAACATGTT	42	2[118]	12[105]
TTTCAACTAATGTGCCTGGAGTGACTCTGGCGAGAAAAACGT	42	16[160]	26[147]
TGCATTAACGGGCAACAAACAATAACGGATTCATTTCCCAGC	42	20[48]	20[49]
AGACTGCGGAACCCCCTCAGAGCCACCGGAGGTTAGCAAAAGACGGAA	48	8[250]	11[244]
GGGTATTATTTTCAGCGAACGAGTAGATGTACCGCACGATAAAAATCAT	49	3[133]	1[153]
TCTCAGATGATGGCATTATTTGCACGTGTTACCTGTTTTTAATGAGCAT	49	25[56]	18[49]
AGGTTATCTAAAATTGAATAACAATCGCAAGACAAAGCAAATAAATATC	49	23[91]	21[111]
TTGCTGATTTTTGCAATTGCTAGACCGGAAGCAAAGTAAGAGGAAGCCC	49	11[105]	7[111]
AACCTCCAGATTAGAGCCATATTTGTTTAGAGAATACAAAGTCAGAGGG	49	2[195]	7[202]
GGAAGAACGCTCGCTAGGTAAAGATTCAGGGAGAAATAAAGCATAAGAA	49	27[161]	18[168]
GAGCGTCATTGACAACCCTCAATAATCAAAATCACTAGCGCGTGAAACC	49	4[237]	9[244]
GTAACCGTCACGTTGTGAGCGAGTAACAATCCTGAGCACCGCTGTTGGG	49	21[238]	18[245]
TTTTCACCCGCCTGTGGTTTGCCCCAGCATCGGCAAGTCGGGAAAGCCT	49	21[28]	18[35]
TGAGTGCCCAGCTATTTGCCAAACACCAGAGCCGCATTACCAGAGGGAG	49	2[216]	11[223]
TTTTTTGAGTGAATAATTTTCTCGGCTGACGCATTGGGGTCGTATTCCT	49	16[83]	26[63]
TAGCGGTCACGCTGTCAAACTTACTTCTCGAGTAACTGTAGCTTAACCA	49	27[203]	22[210]
GTGCCAAGCTGCAAGTGCGGGGCGCAACTTCTGGTACTCCAGGCCAGTT	49	15[252]	21[258]

AAACAGTTAGACTGTGCAACTAAAGTAAGCGCAGACGGTCAGAGCTTAA	49	4[97]	11[104]
ATTACCTAGCAAAAGCGAATTATTCGCCTGATTGCAGTAACAAAAGAAA	49	18[69]	23[76]
ACCCTGAAACATAAAAACAGGATAAGAAAGAACAGTAGGGCTGGTTAAG	49	7[182]	9[174]
ATCGCCATGAATGGTCTGGCCAACAGAGAGATTCAGACCTAATAAACAC	49	21[154]	18[161]
TTCATCGTAGGAACGTACCGCATCGGCTATAATATCCCATCCGAATCGC	49	2[160]	10[154]
GTTTTGCAAAAGAAACAAAAGAAATATTTCACCAGTCACCAATTTTCAT	49	0[237]	8[231]
TAATTTTAGCAGCCTTTACAGAACGTCACCCTTTTACCAGAAGGAAACC	49	7[203]	11[209]
AATTCATGCTACAGATAACGTACAGGAGCGCCAGAACCCGTCCGTAATG	49	14[258]	22[252]
TGTTCCAGGACTCCACACAACCGAGCTCGAATTCGGCTTGTTCCTCCTG	49	25[35]	14[28]
TTCATCGTAGGAACGTACCGCATCGGCTATAATATCCCATCCGAATCGC	49	2[160]	10[154]
GTTTTGCAAAAGAAACAAAAGAAATATTTCACCAGTCACCAATTTTCAT	49	0[237]	8[231]
TAATTTTAGCAGCCTTTACAGAACGTCACCCTTTTACCAGAAGGAAACC	49	7[203]	11[209]
AATTCATGCTACAGATAACGTACAGGAGCGCCAGAACCCGTCCGTAATG	49	14[258]	22[252]
TGTTCCAGGACTCCACACAACCGAGCTCGAATTCGGCTTGTTCCTCCTG	49	25[35]	14[28]
TTCATCGTAGGAACGTACCGCATCGGCTATAATATCCCATCCGAATCGC	49	2[160]	10[154]
GTTTTGCAAAAGAAACAAAAGAAATATTTCACCAGTCACCAATTTTCAT	49	0[237]	8[231]
TAATTTTAGCAGCCTTTACAGAACGTCACCCTTTTACCAGAAGGAAACC	49	7[203]	11[209]
AATTCATGCTACAGATAACGTACAGGAGCGCCAGAACCCGTCCGTAATG	49	14[258]	22[252]
TGTTCCAGGACTCCACACAACCGAGCTCGAATTCGGCTTGTTCCTCCTG	49	25[35]	14[28]
TCAATCGCAGAACACATAAAGTGTAATACTTTTGCAAAGGGTTTCTAAT	49	25[182]	14[175]
CGGAACCACAGGAGCTGAATTAAAGCCAGTCAGACACCGCCAAGAGCCA	49	1[252]	7[258]
AGTGCCTTGCCCGTCAGGCGGATAAGTGCCGTCGATCTAAGAACAACAG	49	2[223]	1[216]
CGGCATTTCTTTTCGAGCCGCCACCAGAACCACATATTAGCGTTTGCCA	49	8[230]	7[223]
CCCTTCACAGTGAGATGAATCGGCCAACGCTTTCCAAATCCCTGAGTGT	49	22[41]	25[34]
TCTCCGAATCACCCAGAACGTGTTTGGATTCCGAAAGGCGAACTGATTG	49	14[48]	22[42]
ATTATTCTACCAGCGCAGTCTTGTACTGGTAATAACCCCCTGTAGCGGG	49	11[245]	0[238]
GCGGCCCACGGGGAGTTTGAGACAACTCAATACATTAATAGATTGGCAA	49	14[132]	22[126]
ATTTTTGTCATTTTCAGCTTTCATCAACATCAGTGTGTCAATATCAGGT	49	21[217]	18[224]
AATGCAGAGATTTAAACTAACAAGAAAAATGCGATAACTTTAAATAAGG	49	1[63]	7[69]
GCGCTAGACCCCGCGAGAAAGGCCGGAGATGACCCCTAAATCAGGTGAT	49	27[182]	18[189]
CAATTCGTAACATTACATCCATCAATAGTGAATTTTTAAGACTGTAAGC	49	25[119]	14[112]
ATAGGGGACTAAATGAAGGAGATCCTGAGTTAGAAGATTTTCTCAGATG	49	14[90]	22[84]
TTAAATAGATAGCGTCCAATACCCTCAACATAAATATCAAAAAGATTAG	49	12[104]	6[98]
GGAGTTATATATTCTTCTTAAACAGCTTTAATTGTAACGAGGTTCCATT	49	8[41]	11[34]
GACAAAATAATTGATAATTTACGAGCAAGAAGCGCATTAGTTTATCAAC	49	9[161]	6[154]
AAACCCTGTTGAAAGGAGCACTAACAACTTGAGGAGAGAAAATAGTAGC	49	21[112]	18[119]
CAGTTTTGTCGTCTATAGAAAGGAACAATGTGTCGGACCCCCAGCGATT	49	2[55]	13[62]
AGAAGGACGGAATATATGGTTATTAAAGTGGGAATGCAGCACTTAGCGTC	50	0[258]	8[251]
**Staples with extension for AuNRs assembly**			
ATGCGTTTAGCTATAAACCAACAAAATA AAAAAAAAAA	38	11[140]	2[140]
CGGAATCAGCAAAAACAGGAAAAGGAAG AAAAAAAAAA	38	18[160]	27[160]
CTAAAGCAAGAAAATGGAAGGTTGTTTG AAAAAAAAAA	38	20[97]	25[97]
CATTATTACAGGTTTCTACCAGTCAGGA AAAAAAAAAA	38	3[77]	4[77]
TCCCAATGAAGCCTTAAATCACGACTTG AAAAAAAAAA	38	4[181]	2[182]
AATAAGAAACAACGAAGAAAAGTCTTTC AAAAAAAAAA	38	9[140]	4[140]
CGCAGAGGAAGATGTATCAAAAATTCAT AAAAAAAAAA	38	20[76]	25[76]
AAACAAGGGTTGTATTGCTGGGCAAGTG AAAAAAAAAA	38	18[202]	27[202]
CATTGCCCGGAGAGGAAGAACCGCGTAA AAAAAAAAAA	38	18[223]	27[223]
GGAAGGTGGCGACAATCCTGACGGGGTC AAAAAAAAAA	38	11[224]	2[224]

AAAGTGTAAACCTGTCGTGAAATGGTGGACAAGAG AAAAAAAAAA	45	18[48]	25[55]
ACCCTAGATAAATCATACAGGTTTAAAAAAGCCGG AAAAAAAAAA	45	16[132]	27[139]
TAGAGAGTACCTTTGGATGGCGAATGACATGCTTT AAAAAAAAAA	45	9[91]	4[98]
TACATAAGAAACGCAATAATATTTGCACGAGGCGT AAAAAAAAAA	45	13[210]	2[203]
ACAATTTCATTTGACAATATAATCATCAAGGTGCC AAAAAAAAAA	45	18[83]	27[76]
GAGGATTTTATCTTACCGAAGAAAATGAGTTACAA AAAAAAAAAA	45	11[210]	4[203]
AATTGAGCAGAAGAGGTTTGATTATTTATTGACGC AAAAAAAAAA	45	20[188]	25[181]
CAATTCTTTTGGGGCGCGATGATCGTCACAAAAGA AAAAAAAAAA	45	13[126]	2[119]
CAAAATAGGCGCTGTAATATCTCAAATTAACCGTT AAAAAAAAAA	45	14[195]	25[202]
AAATAAGGCGTTAACTCAGAGATATTACGGAGCGG AAAAAAAAAA	45	18[188]	27[181]
AACTCGTGAGCCCCTGCGGAACTAGACTTTACAAA AAAAAAAAAA	45	14[111]	25[118]
CGGGTACATACGAGCCGGAGACTATTAAAAATCAA AAAAAAAAAA	45	16[48]	27[55]
CCCAATAATAAGAGAGTAAGCACGATTTTTATTTA AAAAAAAAAA	45	9[175]	4[182]
GATAGCCACACCGCCTGCAACAAATATAAATAGATGTATTAA AAAAAAAAAA	52	21[140]	25[139]
AGATGAACCGCGACCTGCTCCGATAAATTAAAACGGGAATAC AAAAAAAAAA	52	10[55]	2[56]
AGTTTCATCAGAGCCATAGTAAGAGCAACCAGAGGGGGTAAT AAAAAAAAAA	52	13[98]	2[98]
TTAACTAAAGGAATTATCAGCTTGCTAATTACCTTATCTACG AAAAAAAAAA	52	4[55]	4[56]
CAGTATAGCAAATGGAGAACAAGCAATTCCATGTAGAAACCA AAAAAAAAAA	52	11[161]	4[161]
TTAAAGGGAAAAGCCCCAAAAACTAGCAAGGCCACTTGATTA AAAAAAAAAA	52	21[224]	25[223]
ACAAAGTTCAGGGAACGCCAAAAGGAATTGTAGAAAGATTCA AAAAAAAAAA	52	13[77]	2[77]
AGATACATCACCGTCCCAATAGCAAGCATTGCCGTTTTTATT AAAAAAAAAA	52	13[161]	2[161]
TTCGGTCCAAGGCCGGAAACGTAGCACCCGCCAGCTTTCCAG AAAAAAAAAA	52	8[223]	4[224]
ATATGCTGATGCAAATCCAAAATTAATTAGGTTGGACCACCACGGAACC AAAAAAAAAA	59	18[118]	27[97]
CTTCAAATTCAAATGCAAAGCGGATTGCCAAAAATCAGGTCTTATTCAT AAAAAAAAAA	59	8[125]	4[119]
ATATTTTTTAAAAAGAGGTGAGGCGGTCATCTTCTCCAGTCAGCTCATG AAAAAAAAAA	59	22[167]	25[160]
GATTATACAAAGAAGTTATATAACTATATGTAATAATCATTTCGATTTA AAAAAAAAAA	59	25[98]	27[118]
**Staples with extension for pH responsive triplex lock**			
ATACCAAATCGCCTATGTTACCTTTGAATCAAGAGAAATCAA-tttt-duplex 1	66	13[63]	8[63]
duplex 2-tttt-CGTAACAAAGCTGGCTGGCTGACCTTCAAGAGGAC	59	8[62]	10[56]
TGAGAGTTCGTAAAACAGGAAGCATTAA-tttt-ssDNA	52	18[223]	21[216]

Table S2: Sequences of the pH-sensitive DNA locks.

40% TAT	duplex 1	5′ TCCTCCTCCTCTCTCCTCTC 3′
	duplex 2	5′ GAGAGGAGAGAGGAGGAGGA 3′
	ssDNA	5′ CTCTCCTCTCTCCTCCTCCT 3′

Table S5: Thermal annealing temperatures and times.

Temperature, ºC	Time (min)
80	15
79, 78,.., 72, 71	1
70, 69, 68	5
67, 66, 65, 64, 63	10
62	15
61	20
60	30
59, 58,...,39, 38	45
37, 36	30
35	20
34	10
33, 32	5
31, 30,.., 26, 25	2
20	Hold

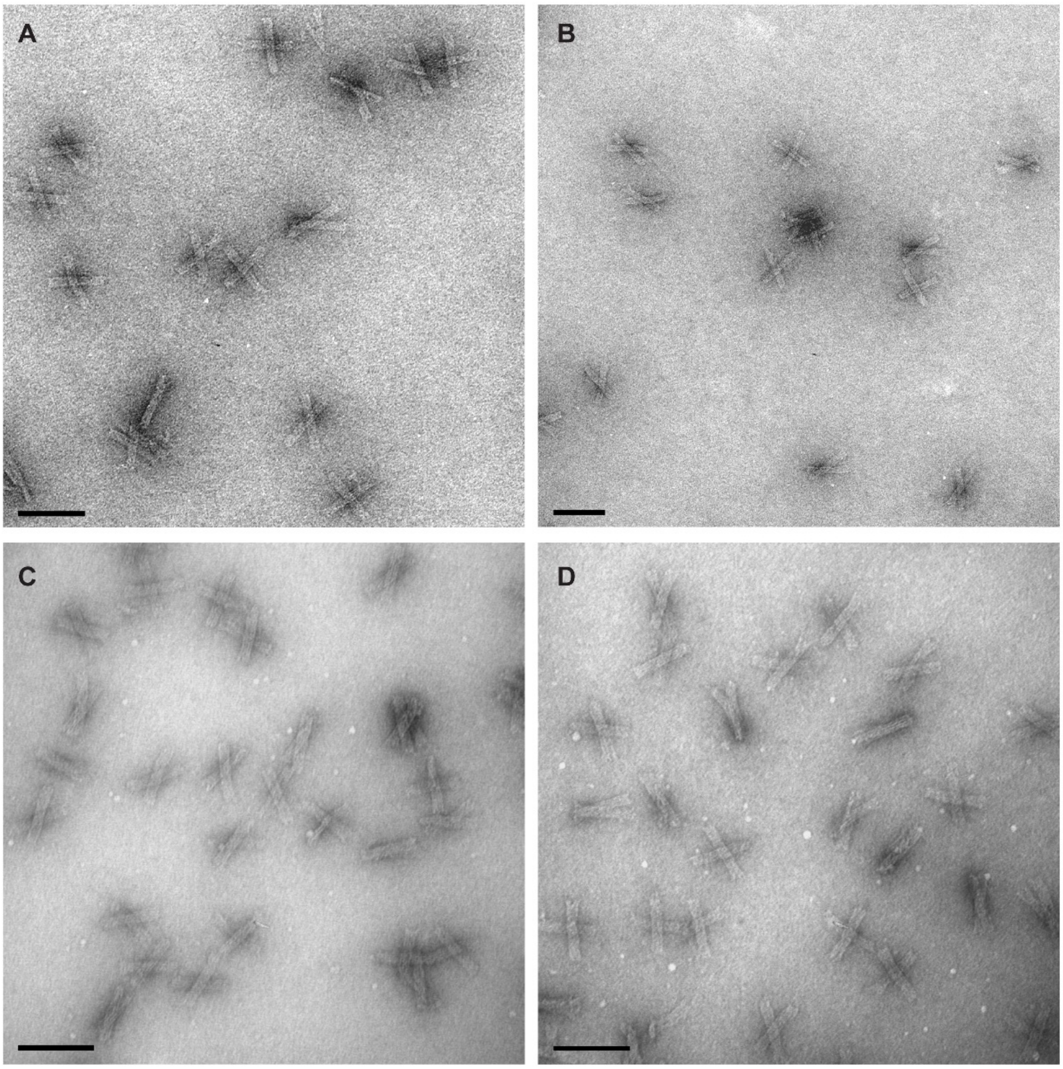

Figure S2. (A, B) TEM micrographs of the DNA origami structures after thermal annealing and purification (0.5× TBE, 10 mM $MgCl_2$). (C, D) TEM micrographs of the DNA origami structures in magnesium free buffer (0.5× TBE, 500 mM NaCl). Scale bars, 100 nm.

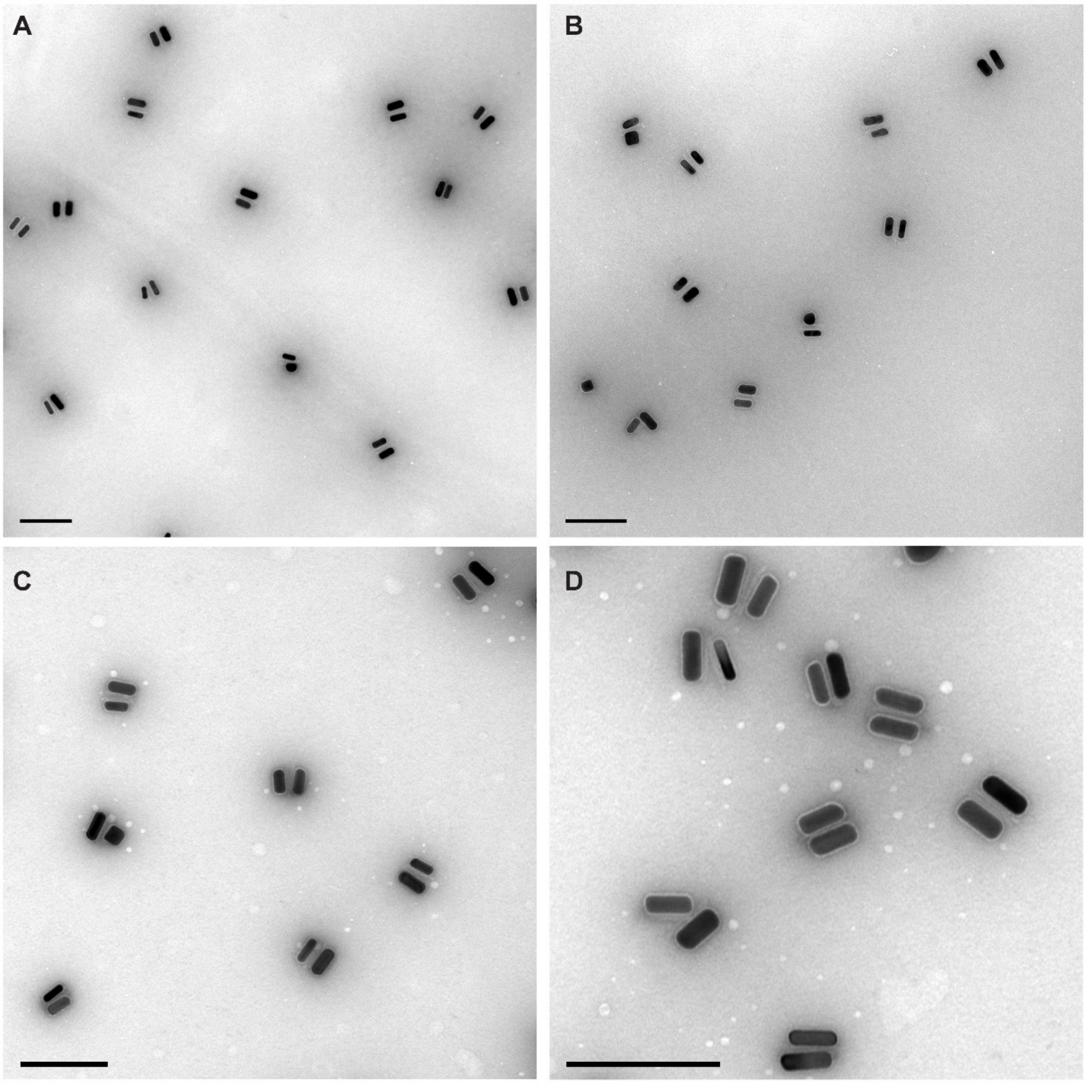

Figure S3. (A, B) Additional TEM micrographs of the CPMs after assembly and purification (0.5× TBE, 10 mM $MgCl_2$, 0.02% SDS). (C, D) TEM micrographs of the CPMs in magnesium free buffers with pH 5.5 (0.5× TBE, 500 mM NaCl, 0.02% SDS, pH adjusted with acetic acid). Scale bars, 200 nm.

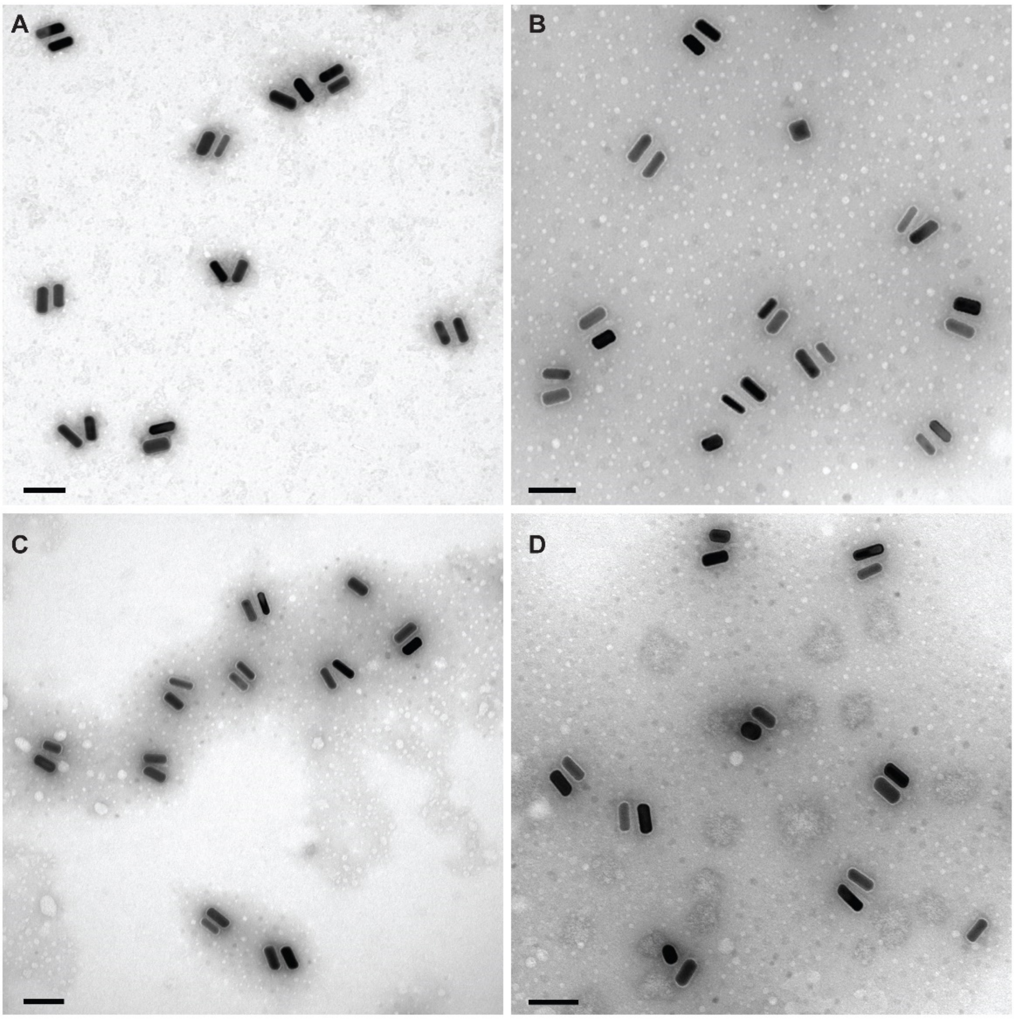

Figure S4. (A, B) TEM micrographs of CPMs in photoresponsive medium (1 mM MCH^+, 0.8% DMSO, 500 mM NaCl, 1 mM $NaHCO_3$, and 0.02% SDS) before illumination. (C, D) TEM micrographs of CPMs in photoresponsive medium after five cycles of illumination (intensity 1.7 mW·cm^{-2}). Scale bars, 100nm

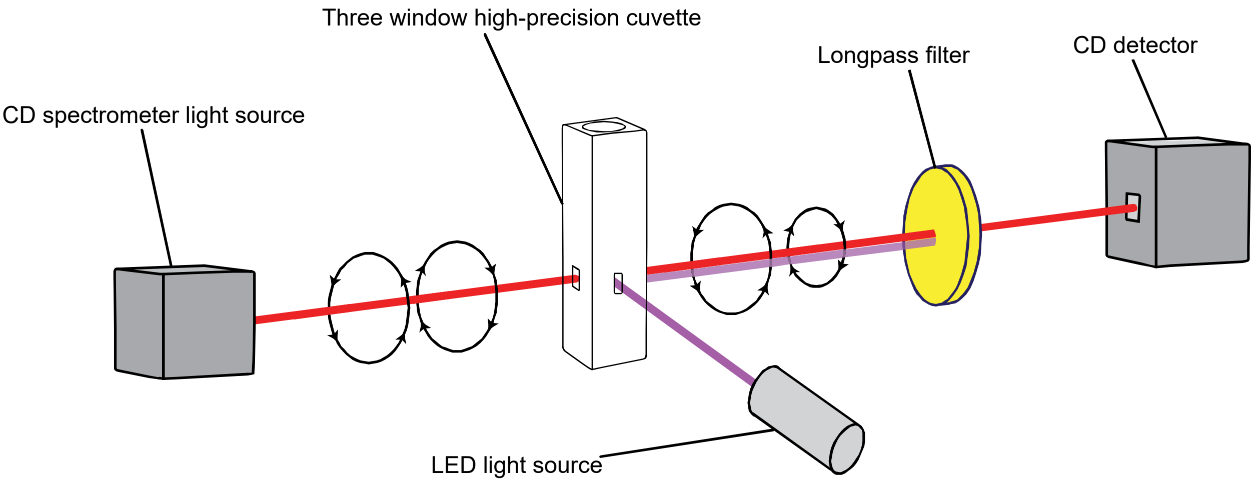

Figure S5. Schematic representation of the experimental measurement setup.

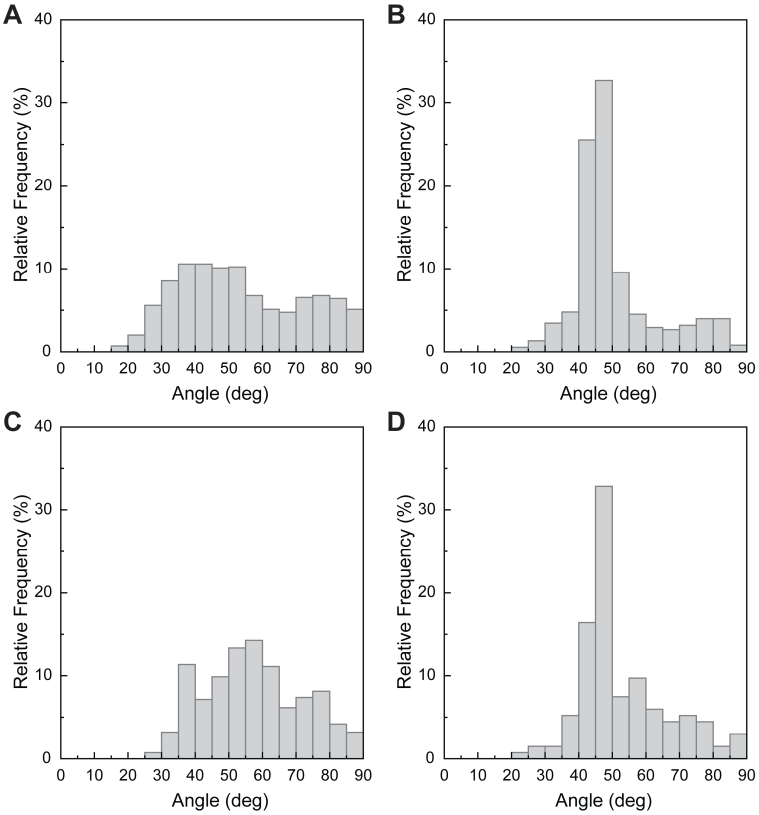

Figure S6. Histograms of the acute angle between two bundles of DNA origami templates. (A) Origami structures deposited on a TEM grid from solution with pH 6.7 (0.5× TBE, 500 mM NaCl, pH adjusted with acetic acid). (B) Origami structures deposited on a TEM grid from solution with pH 5.5 (0.5× TBE, 500 mM NaCl, pH adjusted with acetic acid). (C) Origami structures deposited on a TEM grid from photoresponsive medium without light illumination. (D) Origami structures deposited on a TEM grid from photoresponsive medium under light illumination (intensity 1.7 mW·cm^{-2}). The number of analysed structures: 841 (A), 376 (B), 406 (C), 137 (D). For examples of TEM images used for angle characterization see Figures S7 and S8.

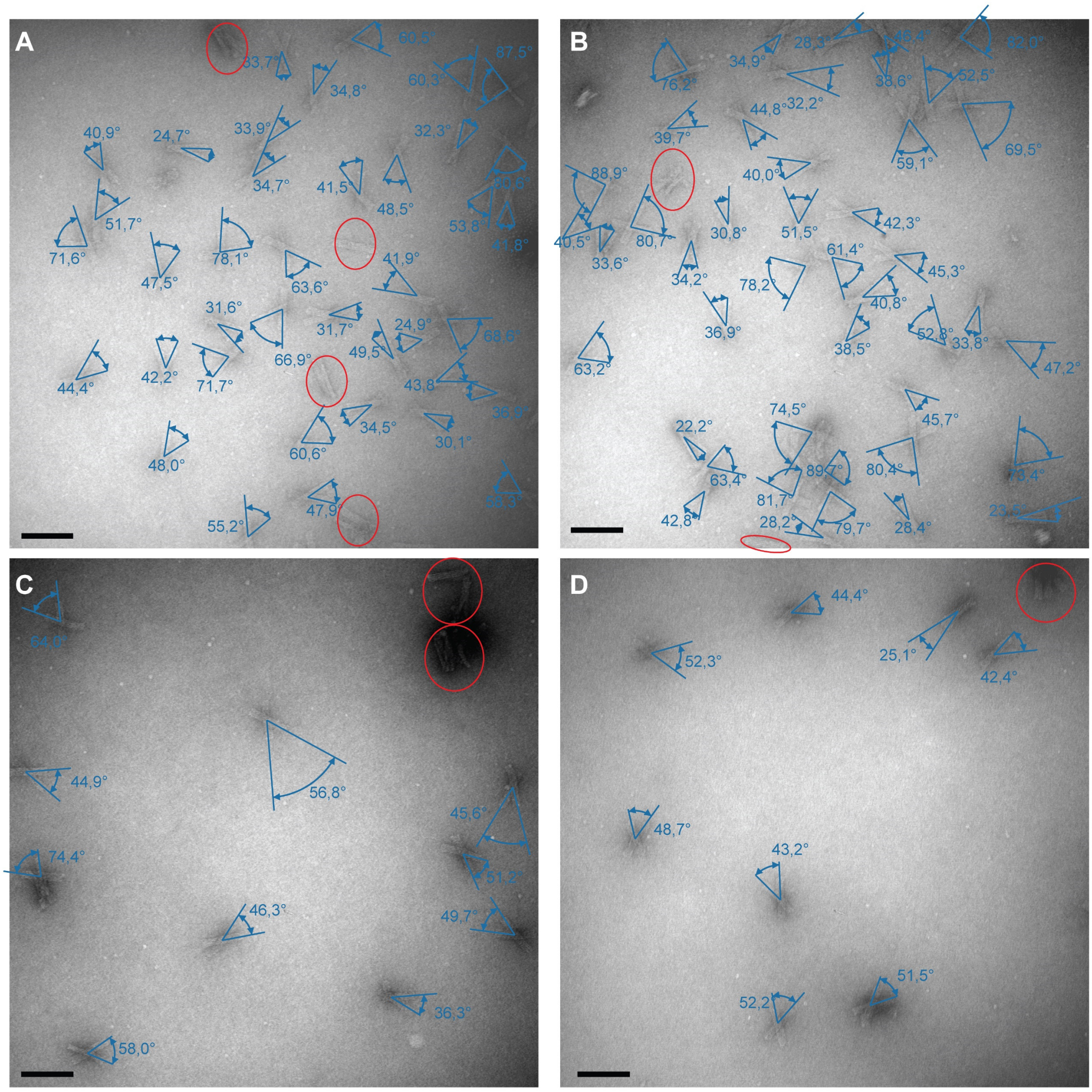

Figure S7. Examples of the acute angle characterization. (A, B) Origami structures deposited on a TEM grid from solution with pH 6.7 (0.5× TBE, 500 mM NaCl, pH adjusted with acetic acid). (C, D) Origami structures deposited on a TEM grid from solution with pH 5.5 (0.5× TBE, 500 mM NaCl, pH adjusted with acetic acid). Structures marked with red ellipses were excluded from analysis. Scale bars, 100 nm.

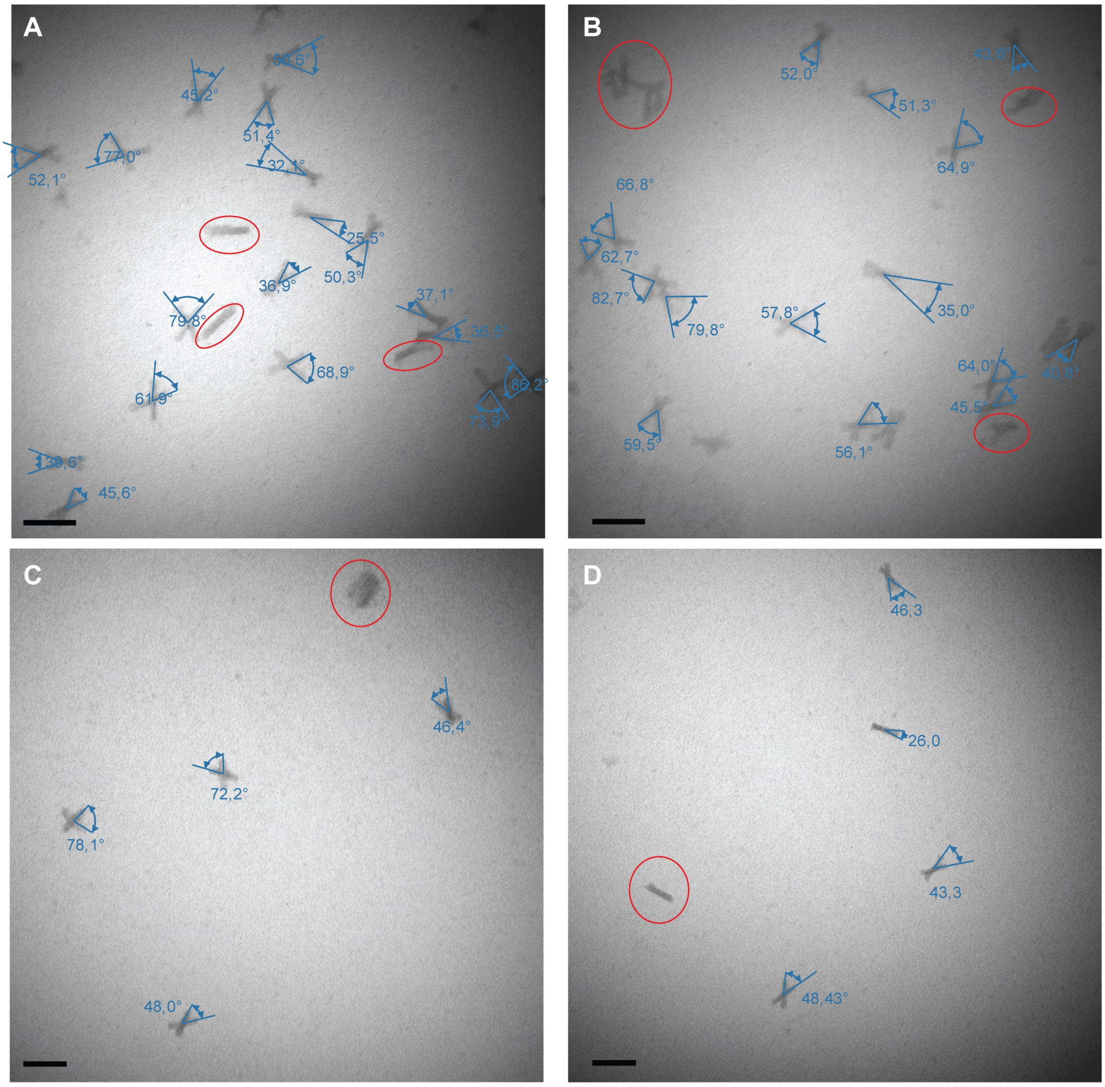

Figure S8. Examples of the acute angle characterization. (A, B) Origami structures deposited on a TEM grid from photoresponsive medium without light illumination. (C.D) Origami structures deposited on a TEM grid from photoresponsive medium under light illumination (intensity 1.7 mW·cm^{-2}). Structures marked with red ellipses were excluded from analysis. Scale bars, 100 nm.

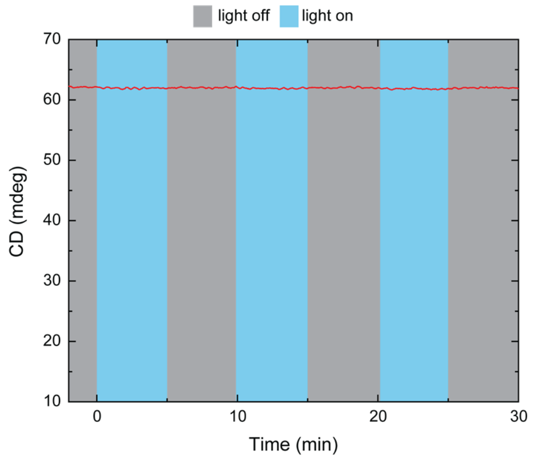

Figure S9. CD responses of right-handed CPMs with pH-insensitive locks (measured at 680 nm) under light illumination (intensity 1.7 mW·cm^{-2}) and in the dark.

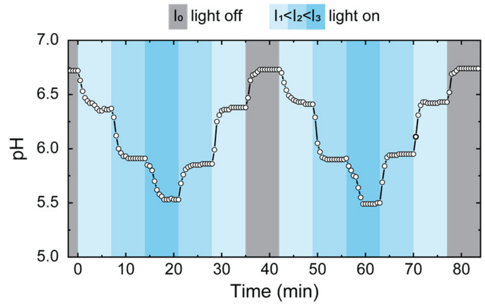

Figure S10. Intensity-dependent modulation of the photoresponsive medium pH. Here, pH value adopted four distinct levels, depending on the intensity of the incident light (I_0 = 0 mW·cm^{-2}, I_1 = 0.21 mW·cm^{-2}, I_2 = 1.3 mW·cm^{-2}, and I_3 = 1.7 mW·cm^{-2}). The switching took ~5 min.

References

[1] A. Kuzyk, M. J. Urban, A. Idili, F. Ricci, N. Liu, *Science Advances* **2017**, *3*, e1602803.
[2] Y. Huang, M.-K. Nguyen, A. K. Natarajan, V. H. Nguyen, A. Kuzyk, *ACS Appl. Mater. Interfaces* **2018**, *10*, 44221–44225.
[3] Y. Huang, M.-K. Nguyen, A. Kuzyk, *JoVE (Journal of Visualized Experiments)* **2019**, e59280.
[4] A. Shaw, E. Benson, B. Högberg, *ACS Nano* **2015**, *9*, 4968–4975.
[5] D. Samanta, D. Galaktionova, J. Gemen, L. J. W. Shimon, Y. Diskin-Posner, L. Avram, P. Král, R. Klajn, *Nature Communications* **2018**, *9*, 641.

Author Contributions

J.R., A.K., and R.K. conceived the research. J.R. and A.K. designed the experiments. A.K. designed the DNA origami structures. J.W. synthesized the photoacid. J.R., A.K.N., and A.J.L. fabricated DNA origami-based chiral plasmonic assemblies and performed the TEM characterizations. J.R. performed the CD characterizations. M.-K.N. fabricated gold nanorods. J.R., A.K., and R.K. wrote the manuscript. All authors have given approval to the final version of the manuscript.
